# Efficacy and Safety of Cefmetazole for Bacteremia Caused by Extended-Spectrum β-Lactamase–Producing Enterobacterales vs Carbapenems: A Retrospective Study

**DOI:** 10.1093/ofid/ofad502

**Published:** 2023-10-07

**Authors:** Eriko Kashihara, Ryuichi Minoda Sada, Yukio Tsugihashi, Hitoshi Obayashi, Akihiro Nakamura, Noriyuki Abe, Hirofumi Miyake, Hiroyuki Akebo

**Affiliations:** Department of General Internal Medicine, Tenri Hospital, Nara, Japan; Department of General Internal Medicine, Tenri Hospital, Nara, Japan; Department of Infection Control, Graduate School of Medicine, Osaka University, Osaka, Japan; Department of Transformative Protection to Infectious Disease, Graduate School of Medicine, Osaka University, Osaka, Japan; Medical Home Care Center, Tenri Hospital Shirakawa Branch, Nara, Japan; Tenri Institute of Medical Research, Nara, Japan; Department of Clinical Laboratory Science, Faculty of Health Care, Tenri University, Tenri, Japan; Department of Clinical Microbiology, Clinical Laboratory Medicine, Tenri Hospital, Tenri, Japan; Department of General Internal Medicine, Tenri Hospital, Nara, Japan; Department of General Internal Medicine, Tenri Hospital, Nara, Japan

**Keywords:** bacteremia, carbapenem, cefmetazole, extended-spectrum β-lactamase–producing Enterobacterales, sequence type 131

## Abstract

**Background:**

Extended-spectrum β-lactamase (ESBL)–producing Enterobacterales have become a global concern owing to increased infections, high mortality, and limited antibiotic treatment options. Carbapenems (CPMs) are effective against ESBL-producing Enterobacterales, but their overuse leads to the emergence of multidrug-resistant bacteria. Cefmetazole (CMZ) is effective in vitro; however, its clinical efficacy remains unclear.

**Methods:**

We retrospectively reviewed patients who were treated with CMZ or CPMs for bacteremia caused by ESBL-producing Enterobacterales between 1 April 2014 and 31 September 2022 at Tenri Hospital. The primary outcome measure was 90-day mortality. We also evaluated resistance genes and sequence types of ESBL-producing Enterobacterales.

**Results:**

In total, 156 patients were enrolled in this study. Ninety patients (58%) received CMZ therapy. Patients in the CMZ group were significantly older than those in the CPM group (median [IQR], 79 years [71–86] vs 74 years [64–83]; *P* = .001). The severity of the Pitt bacteremia score of the CMZ group was lower than that in the CPM group (0 [0–2] vs 2 [0–2], *P* = .042). Six patients (7%) in the CMZ group and 10 (15%) in the CPM group died by day 90 (*P* = .110). Charlson Comorbidity Index and prevalence of sequence 131 between the groups were statistically insignificant.

**Conclusions:**

Our findings suggest that CMZ is a well-tolerated alternative to CPM for treating bacteremia caused by ESBL-producing Enterobacterales.

## Teaser

- Cefmetazole shows potential as an alternative to carbapenem.

The number of extended-spectrum β-lactamase (ESBL)–producing Enterobacterales has increased worldwide in the past 4 decades in community-acquired and nosocomial infections [1]. The 30-day mortality rate due to sepsis caused by ESBL-producing Enterobacterales is as high as about 15%, and the choice of antimicrobial agent significantly affects patient outcomes [2]. The number of antimicrobial agents susceptible to ESBL-producing Enterobacterales is limited, and ESBL-producing Enterobacterales are resistant to most β-lactam and some non–β-lactam antibiotics, including fluoroquinolones and aminoglycosides [3]. Carbapenems (CPMs) are the antimicrobial agents most commonly used worldwide [4]. However, excessive CPM use increases the infection rate of CPM-resistant Enterobacterales [5]. It also poses a serious mortality problem. Falagas et al calculated that 26% to 44% of all-cause deaths among patients with Enterobacterales infection were attributable to CPM resistance [6].

Cephamycins were isolated from Streptomyces in 1972. Cephamycins, such as cefmetazole (CMZ) and flomoxef, fall under the category of second-generation cephalosporins, and their distinction from other second-generation cephalosporins lies in the presence of a methoxy group at the seventh position of cephalosporanic acid. They are resistant to hydrolysis by ESBL-producing Enterobacterales [3] and have good activity against ESBL-producing Enterobacterales in vitro [7]. Cephamycins are widely available and are often used in Japan, Taiwan, and China [8]. However, few studies have compared the effects of cephamycin and CPMs on ESBL-producing infections: most reported a small number of cases, and a few that compared CMZ and CPMs showed patients’ baseline characteristics comparable to those in our study [9–13]. Furthermore, a few articles comparing flomoxef and CPMs for bacteremia caused by ESBL-producing Enterobacterales described the patients’ baseline characteristics, including background disease, but most had small sample sizes [14–16].

The clinical impact of ESBL genes and sequence types has been recently reported [17–19]. Importantly, since 2008, there has been global dissemination of a specific clone of Enterobacterales: *Escherichia coli* sequence type 131 (ST131). This has been associated with the rise in ESBL-producing Enterobacterales [17]. Additionally, it is suggested to be linked to increased mortality [18]. However, limited studies have examined the clinical effect of cephamycins against bacteremia caused by ESBL-producing Enterobacterales. Therefore, the present study aimed to address this knowledge gap by increasing the sample size and examining a more diverse patient population. Furthermore, we aimed to examine the ESBL gene and sequence type distribution and evaluate how gene and sequence type could influence the comparative study between the CMZ and CPM groups.

## METHODS

This retrospective cohort study was conducted at Tenri Hospital, a 715-bed hospital in Nara, Japan. Cases were reviewed for patients who were treated with CMZ or CPMs for bacteremia caused by ESBL-producing Enterobacterales between 1 April 2014 and 31 September 2022. This study followed the STROBE statement (Strengthening the Reporting of Observational Studies in Epidemiology) [20]. The Institutional Review Board of Tenri Hospital approved this study protocol (No. 1216), and the requirement for informed consent was waived.

The criteria for screening ESBL-producing Enterobacterales were per the methods of the Clinical and Laboratory Standards Institute and the European Committee on Antimicrobial Susceptibility Testing: minimal inhibitory concentration ≥8 μg/mL for cefpodoxime or ≥2 μg/mL for ceftazidime, aztreonam, cefotaxime, or ceftriaxone for *E coli*, *Klebsiella pneumoniae*, and *Klebsiella oxytoca*; minimal inhibitory concentration ≥2 μg/mL for cefpodoxime, ceftazidime, or cefotaxime for *Proteus mirabilis* [21]. ESBL production was confirmed with a double-disk synergy test with cefotaxime, ceftriaxone, cefepime, and an amoxicillin-clavulanate disk [22].

We included patients who were >18 years of age, had at least 1 episode of monomicrobial bacteremia caused by ESBL-producing Enterobacterales, and had been treated with CMZ or CPMs as definitive therapy within 3 days after antimicrobial susceptibility data were identified. We excluded patients who had ESBL-producing Enterobacterales resistant to either CMZ or CPMs; a history of an allergic reaction to CMZ, CPMs, or other products in the same class; and pregnancy, lactation, or intentions to become pregnant during the study. ESBL-producing Enterobacterales were defined as positive strains in the double-disk synergy set. The following data were collected:

- Patient characteristics: age, sex, height, body weight, department in which patients were admitted (in our hospital, the Department of General Internal Medicine admits patients with renal and collagen disease), and immunocompromised status
- Charlson Comorbidity Index, community-acquired or nosocomial infection, origin of bacteremia, causative pathogens, and Pitt bacteremia score (PBS)
- The treatment profile outlines the antimicrobials used within 72 hours and the application of drainage therapy

We classified patients as immunocompromised if they met any of the following criteria: taking prednisone >2 mg/kg or >20 mg daily for at least 14 days, having received a biological agent in the previous 30 days, having a history of solid organ transplant, undergoing a hematopoietic stem cell transplant within the past year, receiving cancer chemotherapy within the last 6 months, possessing any congenital immunodeficiency, or living with HIV with a CD4 count of <200 cells/μL [23].

The primary outcome measure was 90-day all-cause mortality. Secondary outcomes included 30-day mortality, recurrent episodes of bacteremia, readmission within 30 days, duration of antibiotic use, and adverse events. Furthermore, we identified the resistance genes of ESBL-producing Enterobacterales. Bacterial DNA was purified with the QIAmp DNA Mini Kit (Qiagen). For *E coli* ST131 typing, ST131 was defined by polymerase chain reaction detection of ST131-specific single-nucleotide polymorphisms in the *mdh* and *gyr*B alleles [20]. In β-lactamase gene typing, strains were analyzed to determine the presence of ESBL encoded by *bla*_CTX-M-1_, *bla*_CTX-M-2_, and *bla*_CTX-M-9_ [24, 25].

### Statistical Analysis

We compared the baseline characteristics of the patients in the CMZ and CPM groups. In addition, we divided the patients into ST131 and non-ST131 groups and compared them with the CMZ and CPM groups. We excluded missing data and used data sets for which all the variables were available. Patient characteristics were described by median (IQR) for continuous variables and number (percentage) for categorical variables. A Mann-Whitney *U* test was used to compare continuous variables between the groups, whereas a Fisher exact test was used to compare categorical variables. The condition for statistical significance was defined as *P* < .05. Statistical analyses were performed with SPSS version 22 (IBM).

## RESULTS

### Patient Characteristics

**Figure 1.**
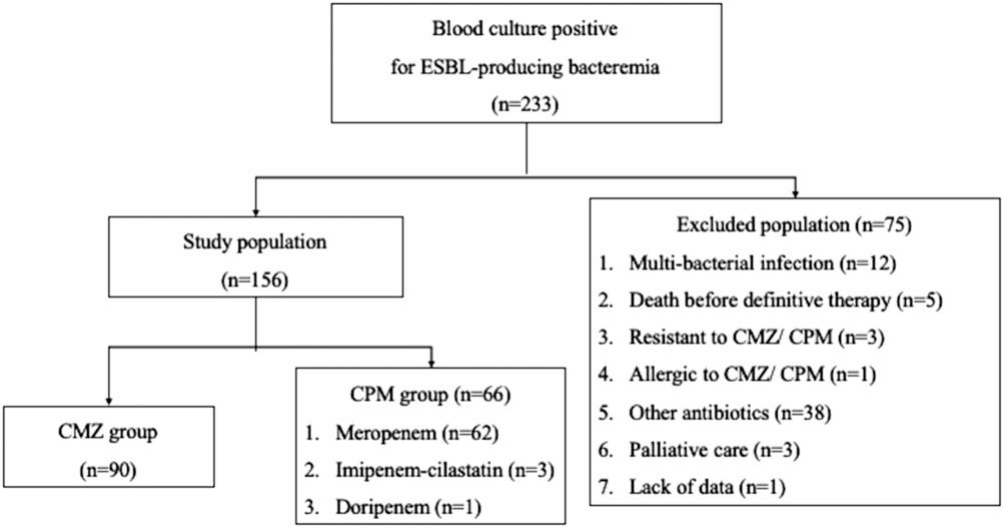
Enrollment of the patients. CMZ, cefmetazole; CPM, carbapenem; ESBL, extended-spectrum β-lactamase.

A total of 233 patients had positive blood cultures for bacteremia caused by ESBL-producing Enterobacterales, of which 156 were included in the analysis. Among them, 90 (58%) received CMZ therapy while 66 (42%) received CPM. Among the CPM group, meropenem was used in 62 patients, followed by imipenem-cilastatin in 3 and doripenem in 1 (Figure 1). In all cases, the doses of antimicrobials were adjusted by type of drug and renal function. The characteristics of the patients are shown in Table 1. Patients in the CMZ group were significantly older than those in the CPM group (median [IQR], 79 years [71–86] vs 74 years [64–83]; *P* = .011), and there were significantly more females in the CPM group (48 [52%] vs 16 [24%], *P* = .010). The severity of the PBS in the CMZ group was significantly lower than that in the CPM group (median [IQR], 0 [0–2] vs 2 [0–2]; *P* = .042). Moreover, no patients in the CMZ group were admitted to the Department of Hematology, and the CPM group had significantly more patients who received a hematopoietic stem cell transplant in the preceding year or cancer chemotherapy within 6 months. However, there were no significant differences in the number of patients who received prednisone ≥2 mg/kg or ≥20 mg daily for at least 14 days or biological agents in the preceding 30 days; in addition, analysis of the other factors, including the Charlson Comorbidity Index, revealed no statistical differences.

**Table 1.** Baseline Characteristics of the Patients

	Patients, Median (IQR) or No. (%)		
	CMZ (n = 90)	CPM (n = 66)	*P* Value
Age, y	79 (71–86)	74 (64–83)	.011
Sex: male	424 (48)	50 (76)	.014
Body mass index	20.3 (18–24)	20.3 (18–24)	.689
Department			
General internal medicine	38 (42)	13 (20)	.003
Urology	19 (21)	6 (9)	.049
Hematology	1 (1)	15 (23)	<.001
Gastroenterology	9 (10)	7 (11)	>.99
Gastrointestinal surgery	1 (1)	6 (9)	.042
Patient profile			
Hospital acquired	22 (24)	29 (44)	.015
Charlson Comorbidity Index	2 (1–4)	3 (2–6)	.069
Pitt bacteremia score	0 (0–2)	2 (0–2)	.042
Underlying disease			
Myocardial infarction	12 (13)	8 (12)	>.99
Congestive heart failure	18 (20)	12 (18)	.840
Peripheral vascular disease	6 (7)	6 (9)	.560
Cerebrovascular disease	17 (19)	12 (18)	>.99
Dementia	22 (24)	14 (21)	.704
Chronic obstructive pulmonary disease	4 (4)	4 (6)	.721
Connective tissue disease	13 (14)	9 (14)	>.99
Peptic ulcer disease	3 (3)	2 (3)	>.99
Liver cirrhosis	6 (7)	6 (9)	.556
Diabetes mellitus			
Uncomplicated	23 (26)	19 (29)	.587
With end-organ damage	8 (9)	9 (14)	.436
Chronic kidney disease	29 (32)	22 (33)	.864
Solid tumor	16 (18)	9 (14)	.659
Leukemia	1 (1)	12 (18)	<.001
Lymphoma	1 (1)	2 (3)	.572
AIDS	0 (0)	0 (0)	. . .
Immunocompromised host	16 (18)	25 (38)	.006
Prednisone >2 mg/kg or 20 mg for at least 14 d	10 (11)	14 (21)	.115
Biological agents in the preceding 30 d	4 (4)	6 (9)	.325
Any history of solid organ transplant	0 (0)	0 (0)	
Hematopoietic stem cell transplant in the preceding year	0 (0)	5 (8)	.012
Cancer chemotherapy within 6 mo	3 (3)	11 (17)	.008
Congenital immunodeficiency	0 (0)	1 (2)	.423
HIV with a CD4 count < 200/μL	0 (0)	0 (0)	
Bacterial profile			
History of ESBL-producing bacteria detection	12 (13)	13 (20)	.377
Pathogen			
* Escherichia coli*	81 (90)	53 (80)	.105
* Klebsiella pneumoniae*	6 (7)	10 (15)	.110
* P mirabilis*	2 (2)	2 (3)	>.99
* K oxytoca*	0 (0)	1 (1)	.423
Device			
Urinary device	14 (15)	19 (14)	.822
Central venous catheter	2 (2)	9 (13)	.009
Endotracheal tube	3 (3)	1 (2)	.64
Prosthetic valve/intracardiac implantable device	5 (6)	5 (8)	.743
Intracranial catheter	0 (0)	0 (0)	
Chest tube	0 (0)	1 (2)	.423
Percutaneous endoscopic gastrostomy	1 (1)	3 (5)	.311
Source			
Urinary	72 (80)	30 (45)	<.001
Abdominal	11 (12)	7 (11)	.805
Respiratory	0 (0)	4 (6)	.030
Catheter-related bloodstream infection	0 (0)	2 (3)	.177
Skin/soft tissue	2 (2)	4 (6)	.403
Bone/arthritis	1 (1)	0 (0)	>.99
Bacteremia with unknown origin	4 (4)	19 (29)	<.001
Treatment profile			
Antibiotics used within 72 h			
Same as the definitive therapy	16 (18)	5 (8)	.095
CPM before CMZ and the opposite	3 (3)	0 (0)	.263
Other active agent	0 (0)	5 (8)	.012
Nonactive agent	71 (79)	56 (84)	.408
Drainage of specific source of infection	19 (21)	14 (21)	>.99

Abbreviations: CMZ, cefmetazole; CPM, carbapenem; ESBL, extended-spectrum β-lactamase.

### Primary and Secondary Outcomes

Six patients (7%) in the CMZ group and 10 (15%) in the CPM group died by day 90 (*P* = .11). Four patients (4%) in the CMZ group and 6 (9%) in the CPM group had recurrent episodes of bacteremia (*P* = .325). Two patients (2%) in the CMZ group and 12 (18%) in the CPM group were readmitted within 30 days of discharge (*P* = .002). The median duration of antibiotic use was 14 days (IQR, 10–14) in the CMZ group and 14 days (11–17) in the CPM group (*P* = .092). Two patients (2%) in the CMZ group and 1 (2%) in the CPM group had adverse events (*P* > .99). The primary and secondary outcomes are shown in Table 2.

**Table 2.** Primary and Secondary Outcomes

	Patients or No. (%)		
	CMZ (n = 90)	CPM (n = 66)	*P* Value
Primary outcome			
90-d mortality	6 (7)	10 (15)	.110
Secondary outcomes			
30-d mortality	2 (2)	5 (8)	.134
Recurrent episode of bacteremia	4 (4)	6 (9)	.325
Readmission within 30 d	2 (2)	12 (18)	.002
Duration of antibiotic treatment, d	14 (10–14)	14 (11–17)	.092
Adverse event	2 (2)	1 (2)	>.99
Allergy	1 (1)	0 (0)	>.99
*Clostridium difficile* infection	1 (1)	1 (2)	.419

Abbreviations: CMZ, cefmetazole; CPM, carbapenem; ESBL, extended-spectrum β-lactamase.

### Microbiological Characteristics

The CMZ and CPM groups had antimicrobial resistance genes such as *bla*_CTX-M-1-like_ (36%, 23%), *bla*_CTX-M-2-like_ (2%, 3%), and *bla*_CTX-M-9-like_ (61%, 71%). Patients who died within 90 days harbored *bla*_CTX-M-1-like_ (n = 3, 4) and *bla*_CTX-M-9-like_ (n = 3, 6) genes. The analysis of ESBL gene types is presented in Table 3. ST131 clones were detected in 66 patients (73%) in the CMZ group and 43 (65%) in the CPM group. There were no significant differences in the prevalence of ST131 between the groups (*P* = .293). In the cohort with non-ST131 clones, no patients in the CMZ group and 3 in the CPM group died within 90 days. We conducted a subgroup analysis of the primary and secondary outcomes of ST131 and non-ST131 clones (Table 4). While hospital-acquired infections, immunocompromised hosts, and PBS were significantly higher in the CPM group of ST131 clones (*P* = .013, *P* = .029, *P* = .020, respectively), there was no significant difference in the primary outcome between the CMZ and CPM groups in either cohort. Among the ST131 clones, there were significantly more patients readmitted within 30 days in the CPM group than the CMZ group (*P* < .001), while non-ST131 clones did not demonstrate significant differences.

**Table 3.** Analysis of ESBL Genogroups

	ESBL Gene Type, No.^a^								
	*bla*_CTX-M-1-like_			*bla*_CTX-M-9-like_			*bla*_CTX-M-2-like_ + *bla*_CTX-M-9-like_		
	CMZ (n = 32)	CPM (n = 15)	*P* Value	CMZ (n = 55)	CPM (n = 47)	*P* Value	CMZ (n = 1)	CPM (n = 0)	*P* Value
Mortality									
90 d	3	4	.188	3	6	.295	0	0	. . .
30 d	1	2	.235	1	3	.332	0	0	. . .
Bacteremia recurrence	3	3	.367	1	3	.332	0	0	. . .
Readmission within 30 d	0	2	.097	2	10	.011	1	0	. . .

Abbreviations: CMZ, cefmetazole; CPM, carbapenem; ESBL, extended-spectrum.

^a^Note that 2 gene sets had zero cases of mortality, recurrence, or readmission: *bla*_CTX-M-2-like_ (CMZ, n = 2; CPM, n = 2) and *bla*_CTX-M-1-like_*+ bla*_CTX-M-2-like_ (CMZ, n = 0; CPM, n = 2),

β-lactamase.

**Table 4.** Subgroup Analysis of Sequence Types

	Sequence Type, Median (IQR) or No.					
	ST131 (n = 109)			Non-ST131 (n = 47)		
	CMZ (n = 66)	CPM (n = 43)	*P* Value	CMZ (n = 24)	CPM (n = 23)	*P* Value
Age, y	79 (70–86)	74 (64–84)	.101	78 (73–80)	74 (66–80)	.034
Sex: male	31	29	.049	11	21	.149
Patient profile						
Hospital acquired	15	21	.013	6	8	.534
Charlson Comorbidity Index	2 (1–4)	3 (2–6)	.134	2 (1–4)	3 (2–4)	.311
Pitt bacteremia score	0 (0–2)	2 (0–3)	.02	2 (0–2)	2 (0–2)	.122
Immunocompromised host	9	14	.029	7	11	.238
Bacterial profile						
History of ESBL-producing bacteria detection	8	6	.778	4	7	.318
Pathogen						
* Escherichia coli*	66	41	.153	15	12	.561
* Klebsiella pneumoniae*	0	2	.153	6	8	.534
Source						
* *Urinary	52	19	<.001	19	11	.036
Primary outcome						
90-d mortality	6	7	.365	0	3	.109
Secondary outcomes						
30-d mortality	2	5	.110	0	0	
Recurrent episode of bacteremia	2	4	.210	2	2	>.99
Readmission within 30 d	2	10	.001	1	2	.168

Abbreviations: CMZ, cefmetazole; CPM, carbapenem; ESBL, extended-spectrum β-lactamase; ST131, sequence type 131.

## DISCUSSION

To the best of our knowledge, our retrospective study examined the largest sample size to date on patients treated with CMZ or CPMs for bacteremia caused by ESBL-producing Enterobacterales. Furthermore, our study included several patients with hematologic tumors and neutropenic fever, which were not included in previous studies. Our results suggest similar efficacies against bacteremia caused by ESBL-producing organisms. Fukuchi et al reported the efficacy of CMZ as a definitive therapy against bacteremia caused by ESBL-producing Enterobacterales as compared with CPM. Their results revealed no significant differences in 90-day mortality rate between the groups (1/26 vs 5/43) [10]. Matsumura et al showed that the treatment of patients with bacteremia caused by ESBL-producing *E coli* with CMZ or flomoxef was similarly effective as treatment with CPM (30-day mortality was 3/59 vs 5/54, respectively) when the patients did not have hematologic malignancy or neutropenia [13]. Moreover, the number of the adverse events seems to be as small as that in previous studies [10, 13], which might indicate a benefit of the CMZ being with the narrow spectrum. Based on the findings of our study, CMZ emerges as a potential therapeutic option for the management of bacteremia caused by ESBL-producing Enterobacterales in patients with hematologic malignancies and/or neutropenic fever.

Patients with ESBL-producing *K pneumoniae* bloodstream infections have higher mortality than those with ESBL-producing *E coli* [26, 27]. CPMs have been recommended for ESBL-producing *Klebsiella* bacteremia in previous studies [28, 29]. However, there have been limited data on the effectiveness of CMZ for ESBL-producing *K pneumoniae* bacteremia. Two previous reports stated the efficacy of CMZ when compared with CPM. Fukuchi et al reported that the proportion of *Klebsiella* spp was 6% (4/69; 2 cases of *K pneumoniae* and 2 cases of *K oxytoca*) [10], whereas Matsumura et al analyzed the bacteremia caused by *E coli* only [13]. Yet, in our study, the prevalence of *Klebsiella* spp was 11% (18/158 cases; 17 cases of *K pneumoniae* and 1 case of *K oxytoca*). This prevalence is higher than in the previous 2 studies. Moreover, in our study, the mortality rate caused by *Klebsiella* spp bacteremia was 0% (0/8) in the CMZ group and 40% (4/10) in the CPM group. Our study indicated the possible efficacy of CMZ in treating ESBL-producing Enterobacterales, including *Klebsiella* spp.

We analyzed the genotypes and sequence types of ESBL-producing Enterobacterales in the present study. CTX M15 β-lactamases are the most common type of ESBLs identified in Europe and some countries in Asia, Africa, North America, South America, and Australia [30]. Similar to previous ESBL genotyping studies conducted in Japan [31–33], CTX M9 was predominant in our study. When a subanalysis was conducted for each genotype group, there was no notable disparity in primary and secondary outcomes between the CMZ group and the CPM group. This observation aligns with the limited existing studies, which have not consistently reported differences in mortality based on genotype. The prevalence of ESBL-producing ST131 strains has increased rapidly [34]. The prevalence rates of ST131 clones in previous studies (62% and 43%) were not significantly different from those in the present study [30, 31]. The clinical impact of ST131 in patients with bacteremia caused by ESBL-producing Enterobacterales has remained controversial [17], but Wang et al reported that the ST131 clone was associated with a higher 28-day mortality in patients with bacteremia caused by ESBL producers [34]. In this study, ST131 clones might have affected the readmission rate within 30 days, but the presence or absence of ST131 clones did not influence other characteristics and outcomes. The genotype and sequence type results indicated that CMZ was as effective as CPM, regardless of the sequence type.

This study had several limitations. First, this was a retrospective study conducted at a single institution. Second, there was a noticeable difference between the groups in the number of patients who were admitted to the Department of Hematology orwho had received chemotherapy, as well as in PBS result and receipt of bone marrow transplant. Third, some patients were treated with empiric antibiotics before definitive therapy. However, CPM was used before CMZ in only 3 patients (3.3%) in the CMZ group.

## CONCLUSION

The findings of our retrospective study suggest that CMZ is a well-tolerated alternative to CPM for treating bacteremia caused by ESBL-producing Enterobacterales. More extensive prospective studies in multiple settings and multicenter randomized trials are required to corroborate these findings.

## Notes

***Acknowledgments*.** We express our gratitude to the members of the Microbiological Research Centre for their excellent care and assistance. This research was conducted as part of the All-Osaka U Research in “The Nippon Foundation–Osaka University Infectious Disease Response Project.”

***Author contributions***. E. K. and R. M. S. drafted and revised the manuscript and collected the data for this study. A. N. and N. A. conducted microbiological analyses. Y. T., H. O., A. N., N. A., H. M., and H. A. supervised the study and revised the manuscript. All authors have approved the manuscript for submission.

***Availability of data and materials***. Data are available from the authors upon reasonable request and with permission from the institutional review board.
